# Global, regional, and national burden of alopecia areata from 1990 to 2021: An analysis from the Global Burden of Disease Study 2021

**DOI:** 10.1016/j.jdin.2025.11.027

**Published:** 2025-12-17

**Authors:** Dezhao Bi, Songmao Hua, Yunyao Hu, Jinrui Zhang, Haolong Ran, Lei Han, Jin Tong Tey, Shun Guo

**Affiliations:** a Division of Dermatology, Affiliated Hospital of Nanjing University of Chinese Medicine, Jiangsu Province Hospital of Chinese Medicine, Nanjing, China; b The First Clinical Medical College of Nanjing University of Chinese Medicine, Nanjing, China

**Keywords:** alopecia areata, disease burden, epidemiologic study, GBD, public health

*To the Editor:* Alopecia areata (AA) is an autoimmune hair disorder affecting approximately 2% of the global population. Although not life-threatening, it significantly impairs mental health and quality of life.^1^^,^^2^ Using data from the Global Burden of Disease 2021 study, this research systematically analyzes global trends in the epidemiologic burden of AA from 1990 to 2021.

Data on AA incidence, prevalence, disability-adjusted life years (DALYs), and the Socio-demographic Index across 204 countries and territories were extracted from the Global Burden of Disease 2021 database. Joinpoint regression was used to calculate the annual percent change. An age-period-cohort model was applied to examine age, period, and birth cohort effects, and decomposition analysis was employed to identify drivers of changes in DALYs. In addition, information on data definition, statistical modeling, and improving data quality is described in detail.^3^

From 1990 and 2019, the age-standardized incidence rate, prevalence rate, and DALYs for AA showed a declining trend globally, while the absolute numbers demonstrated a clear upward trend (Table I). Decomposition analysis revealed that population growth was the primary driver of the increase in DALYs (accounting for 94.44%), with population aging contributing an additional 14.41%. The disease burden exhibited distinct patterns across different populations: the burden was significantly higher in females than in males (Fig 1), and the risk increased with age, peaking in the 30-34 age group. Geographically, Kuwait experienced the largest increase in the age-standardized incidence rate, whereas the United States Virgin Islands saw the most pronounced decline. Furthermore, an increasing disease burden was observed among the elderly population in high Socio-demographic Index regions. Detailed data are provided in the Supplementary Material, available via Mendeley at https://data.mendeley.com/datasets/dm4bw6nb6z/1.

**Table I:** Global all-age cases and age-standardized incidence rates, prevalence rates, and disability-adjusted life years (DALYs) of alopecia areata (AA) from 1990 to 2019

Global	All ages cases		Age-standardized rates per 100,000 people	
	1990 *n* (95% UI)	2021 *n* (95% UI)	1990 *n* (95% UI)	2021 *n* (95% UI)
Incidence				
Total	20428086 (19765445,21094767)	30893047 (29948975,31819277)	393.69 (381.63,405.69)	379.54 (368,391.05)
Male	7215280 (6964329,7459053)	10737101 (10404194,11075546)	272.41 (263.53,281.3)	263.97 (255.73,272.41)
Female	13212807 (12786869,13636184)	20155946 (19536634,20759058)	513.98 (498.44,530.03)	494.8 (479.11,510.04)
Prevalence				
Total	11526305 (11152046,11891144)	17525630 (16977878,18072092)	223.04 (216.06,230.08)	215.01 (208.32,221.75)
Male	4071483 (3921385,4213170)	6089814 (5893833,6288977)	154.39 (149.12,159.58)	149.54 (144.63,154.42)
Female	7454822 (7211776,7689560)	11435816 (11081037,11783723)	291.04 (281.91,299.8)	280.23 (271.33,288.92)
DALYs				
Total	377772 (244971,535131)	571860 (371725,808502)	7.29 (4.74,10.29)	7.02 (4.56,9.94)
Male	134518 (86920,191449)	200722 (130267,285789)	5.08 (3.29,7.23)	4.93 (3.2,7.03)
Female	243254 (158374,343012)	371138 (242397,522713)	9.47 (6.19,13.32)	9.11 (5.94,12.85)

*DALYS*, Disability-adjusted life years.

**Fig 1:**
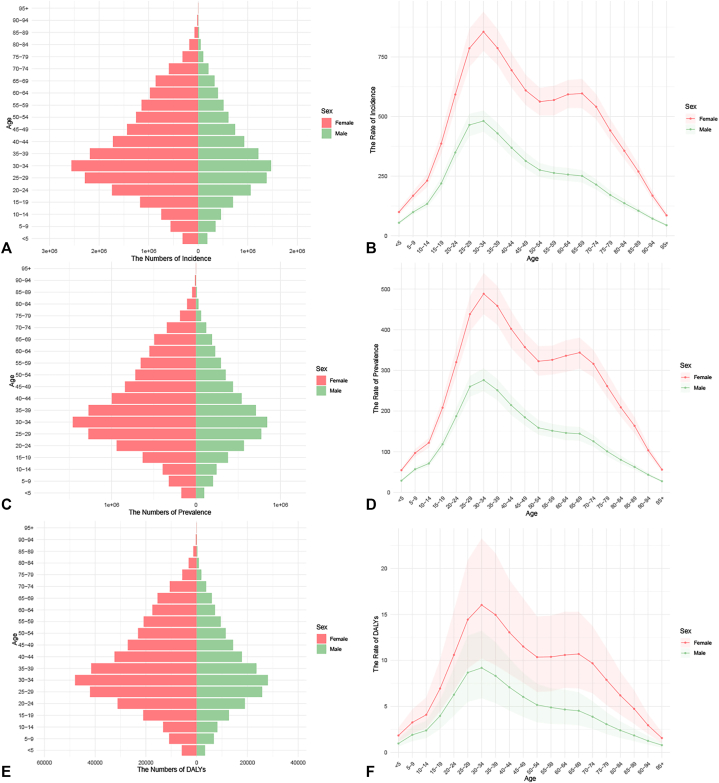
Age-specific numbers and age-standardized incidence, prevalence and DALYs rates of AA in global, 2021. **A,** Age-specific incidence number. **B,** Age-standardized incidence rate. **C,** Age-specific prevalence number. **D,** Age-standardized prevalence rate. **E,** Age-specific DALYs number. **F,** Age-standardized DALYs rate. *DALYS*, Disability-adjusted life years.

Despite declining trends in age-standardized incidence, prevalence, and DALYs rates of AA, the absolute number of cases, prevalence, and DALYs have increased significantly. This growth may be attributed to global population growth—particularly the aging process—as well as environmental changes. As the Socio-demographic Index rises, improvements in life expectancy and quality of life are accompanied by an increasing burden of chronic diseases such as AA. Globally, the disease burden of AA is significantly higher in female than in male. This disparity may be linked to several factors: female generally place greater emphasis on physical appearance, leading to more pronounced psychological stress and social impact from AA; in addition, hormonal changes—such as those during pregnancy and menopause—may influence the course of hair loss in female.^4^^,^^5^ Regardless of gender, the risk of AA peaks between ages 30 and 34. This life stage, often associated with heightened life stress, occupational responsibilities, and changes in family roles, may serve as potential contributing factors.

It should be noted that this study has several limitations. First, there may be constraints in the reliability and completeness of the underlying data. Second, the stratification of AA by severity remains insufficient. Additionally, the Global Burden of Disease analytical model may inadequately account for regional variations in sociocultural and behavioral factors, which could lead to underestimation or overestimation of disease burden in specific populations or geographic areas.

## Conflicts of interest

None disclosed.
